# Diagnostic accuracy of diffusion weighted imaging for differentiation of supratentorial pilocytic astrocytoma and pleomorphic xanthoastrocytoma

**DOI:** 10.1007/s00234-018-2036-y

**Published:** 2018-05-24

**Authors:** Dejun She, Jianyi Liu, Z. Zeng, Z. Xing, Dairong Cao

**Affiliations:** 1 Department of Radiology, First Affiliated Hospital of Fujian Medical University, 20 Cha-Zhong Road, 350005 Fuzhou, Fujian People’s Republic of China; 2 Department of Medical Imaging Technology, College of Medical Technology and Engineering, Fujian Medical University, 350108 Fuzhou, Fujian People’s Republic of China

**Keywords:** Supratentorial pilocytic astrocytoma, Pleomorphic xanthoastrocytoma, Diffusion-weighted imaging

## Abstract

**Purpose:**

Supratentorial pilocytic astrocytoma (PA) may mimic pleomorphic xanthoastrocytoma (PXA) on conventional MR imaging, and a differentiation is clinically important because of distinct recurrence rate and anaplastic transformation rate. The purpose of this study was to investigate the diagnostic potential of diffusion-weighted imaging (DWI) in differentiating supratentorial PA from PXA.

**Methods:**

We retrospectively reviewed DWI and conventional MR imaging of 16 patients with supratentorial PA and 8 patients with PXA. Variables of mean ADC values (ADC_mean_) and minimum ADC values (ADC_min_) were calculated from the ROIs containing the contrast-enhancing lesion on DWI. ADC_mean_ values and ADC_min_ values were compared among all supratentorial PA and PXA as well as between the subgroup of lobar PA and PXA by using an unpaired Student’s *t* test. The optimum threshold, sensitivity, specificity, accuracy, and the area under the receiver operating characteristic curve (AUC) were determined.

**Results:**

Both ADC_mean_ values (1542 ± 186 vs 1084 ± 201 × 10^−6^ mm^2^/s; *P* < 0.001) and ADC_min_ values (1355 ± 183 vs 988 ± 180 × 10^−6^ mm^2^/s; *P* < 0.001) were significantly higher in supratentorial PA compared with PXA. The ADC_mean_ values and ADC_min_ values were also significantly higher in lobar PA than those in PXA. The ADC_mean_ values were useful for differentiating supratentorial PA from PXA, with a threshold value of > 1189.8 × 10^−6^ mm^2^/s (sensitivity, 93.8%; specificity, 100%). The optimal threshold values of > 1189.8 × 10^−6^ mm^2^/s for ADC_mean_ values provide sensitivity and specificity of 85.7 and 100%, respectively, for discriminating lobar PA from PXA. The optimum threshold value for ADC_min_ was > 1063.5 × 10^−6^ mm^2^/s.

**Conclusion:**

DWI is helpful in characterization and differentiation of supratentorial PA from PXA.

**Electronic supplementary material:**

The online version of this article (10.1007/s00234-018-2036-y) contains supplementary material, which is available to authorized users.

## Introduction

Pilocytic astrocytoma (PA) is classified as World Health Organization (WHO) grade I tumor, which occurs most commonly in children and young adults [1]. This tumor usually arises from the cerebellum, but it can also occur in the supratentorial compartment including the optic nerve and chiasm, cerebral hemispheres, hypothalamus, or cerebral ventricles [2]. Pleomorphic xanthoastrocytoma (PXA) is a rare brain tumor and is classified as grade II according to the 2016 WHO classification system [3]. PXA typically occurs in the supratentorial brain, most frequently affecting children and adolescent [4]. Both PA and PXA are potentially curable by total surgical resection and are associated with a longer overall survival [5, 6], but the patients with PXA have a far worse prognosis than those with PA, especially in younger patients [4]. Although PXA is considered a benign tumor, population-based studies suggested that this tumor was associated with higher risk of recurrence and anaplastic transformation compared with PA [1, 5, 7–10]. Thus, the differentiation of PXA from PA is important clinically.

PA could be reliably differentiated from PXA when PA occurs in the cerebellum on conventional MR. Unfortunately, when PA occurs in the supratentorial brain, differential diagnosis of supratentorial PA and PXA can be challenging on conventional MR imaging due to their similar neuroradiological presentations, typically appearing as a large cystic mass with a mural-enhancing nodule [6]. Given that differentiation of supratentorial PA and PXA is challenging to the neuroradiologist, diffusion-weighted imaging (DWI) might complement functional and physiological information in addition to that obtained with the anatomic MRI [11–14]. DWI could noninvasively evaluate the Brownian movement of water molecules and reflect tissue cellularity by apparent diffusion coefficient (ADC) values, which may be helpful in the preoperative diagnosis and grading of brain astrocytoma [11–13, 15]. Although both PA and PXA belong to the same family of low-grade astrocytoma, PA may present with histologic structures different from those found in PXA. Therefore, the application of DWI technique may better evaluate and distinguish the cytostructural differences occurring between PA and PXA.

To date, DWI studies in low-grade glioma and on glioma grading have not reported ADC values of PA separately from PXA [15–17]. The aim of this study was to investigate the diagnostic potential of DWI in differentiating supratentorial PA from PXA.

## Materials and methods

### Patients

This retrospective study was approved by the institutional review board of our hospital, and written informed consent was waived due to its retrospective nature. From March 2010 through October 2017, potentially eligible patients with pathologically confirmed supratentorial PA and PXA were identified. For the selection of appropriate patients, those with obvious hemorrhagic lesions, previously treated, or incomplete DWI raw data were excluded. The patients with pathologically confirmed anaplastic PXA were also excluded. Pretreatment MR images of consecutive patients were reviewed retrospectively, and DWI was requested in addition to conventional MRI.

All patients underwent surgical resection after MR examination. Tumor specimens were fixed in 10% phosphate-buffered formalin, embedded in paraffin, and representative slides were stained with hematoxylin-eosin reagent for standard histological diagnosis based on the histologic features by a neuropathologist (8 years of experience in histology) according to the 2016 WHO classification of tumors of the central nervous system (revised 4th edition) [18].

### Imaging protocols

Imaging was performed with a 3.0 T MR system (Skyra; Siemens, Erlangen, Germany) or another 3.0 T MR system (Magnetom Verio; Siemens, Erlangen, Germany) using a head coil. The conventional MR imaging sequences were performed by using axial T2WI, axial and sagittal T1WI, axial FLAIR, and contrast-enhanced T1WI in 3 orthogonal planes. FOV at 220 mm, section thickness of 5 mm, and intersection gap of 1 mm were uniform in all sequences.

Echo-planar DWI was acquired in the transversal plane with *b* = 0 and 1000 s/mm^2^. Acquisition parameters for the DWI sequence were as follows: repetition time (TR)/echo time (TE), 8200/102 ms; FOV, 220 mm; section thickness, 5 mm; intersection gap, 1 mm; NEX, 2.0. DWI was performed before administration of contrast material. Processing of the ADC map was generated automatically on the MR unit.

### Data processing

An experienced neuroradiologist (D.C., with 25 years of brain MR imaging experience) who was blinded to histopathologic results retrospectively interpreted all MR images. The reader evaluated each lesion and recorded the following MR findings: (a) tumor location, (b) predominant radiologic pattern (a cystic mass with a mural nodule or a predominantly solid mass), (c) peritumoral edema, (d) contrast enhancement pattern (mild enhancement or marked enhancement), (e) presence of adjacent leptomeningeal involvement (dural tail sign), and (f) presence of inner tables scalloping. The tumor location was defined as the main lobe when more than one lobe was involved by the lesion. For evaluation of DWI sequence, qualitative assessment of the DWI signal intensity in the contrast-enhanced solid portions of the lesion was performed. The signal intensity of the lesion was classified as hyperintense, isointense, or hypointense compared with the contralateral normal-appearing white matter on DWI map with *b* = 1000 s/mm^2^.

Another two experienced neuroradiologists (Z.X and J.L, with 8 and 3 years of brain MR imaging experience, respectively) independently and manually placed the region of interest (ROI) on ADC maps to encompass the contrast-enhancing solid portion of the tumors at the representative slice. For each tumor, the contrast-enhancing solid part was identified on contrast-enhanced T1-weighted images and matching ADC maps. The representative slice was defined as containing the largest area of the contrast-enhancing solid portion of each lesion and was selected independently by each observer. To obtain minimum ADC values (ADC_min_), a maximum of 6 ROIs depending on tumor size (range 2–6, size 20–30 mm^2^) were positioned without any overlapping inside the tumors on ADC maps. To obtain mean ADC values (ADC_mean_), freehand ROI was delineated along the border of the contrast-enhancing solid regions of each tumor. Cystic, necrotic, hemorrhagic, or apparent vessel regions that might interfere with ADC values were avoided.

The patients with supratentorial PA were further divided into two subgroups based on the tumor location: lobar PAs were located in the cerebral hemisphere, including the frontal lobe, temporal lobe, occipital lobe and parietal lobe, and other supratentorial PAs were located in the other supratentorial regions, including the suprasellar region, dorsal thalamus, and lateral ventricle.

### Data analysis

All statistical analyses were performed with Statistical Package for the Social Sciences (SPSS 22.0 version for Windows, SPSS Inc., IBM) and MedCalc software (version 17.9.7 for Microsoft Windows 10; MedCalc Software, Mariakerke, Belgium). Results with *P* values less than 0.05 were considered to indicate statistical significance. All parameters were presented as mean ± standard deviation. The demographic data and conventional MRI features of supratentorial PA and PXA were compared by using the Chi-square test. Comparisons of the DWI signal intensity between patients with supratentorial PA and those with PXA were made with the nonparametric Mann-Whitney statistical test. The interobserver variability in determining the ADC parameters by two readers was evaluated by the intraclass correlation test (ICC). If the interobserver variability for ADC parameters has an excellent agreement (ICC > 0.75), the opinions of these two readers were intergraded through taking the average of values. The ADC_mean_ values and ADC_min_ values were compared using the unpaired Student’s *t* test in all supratentorial PA and PXA and then between lobar PA and PXA.

The receiver operating characteristic (ROC) analysis curves were constructed to determine the diagnostic accuracy and optimum threshold value of each ADC parameter for discriminating supratentorial PA, especially lobar PA, from PXA. The optimum threshold value defined was those that provided highest sensitivity and specificity jointly and maximized Youden index based on the decision plot. The sensitivity, specificity, positive predictive value, negative predictive value, accuracy, and area under the curve (AUC) based on optimum cut-off values for each ADC parameter were further calculated. Furthermore, comparisons of AUCs for different ADC parameters were made with a *Z* test.

## Results

Twenty-four pathologically proved cases, including 16 cases with supratentorial PA and 8 cases with PXA, were enrolled in this study. The demographic data and conventional MRI manifestations of supratentorial PA and PXA are summarized and compared in Table 1. There was no imbalance in the baseline clinical features, tumor locations, and conventional MR features between supratentorial PA and PXA.

**Table 1:** The demographic data and conventional MR imaging characteristics of supratentorial PA and PA

	Supratentorial PA (*n* = 16)	PXA (*n* = 8)	*P* value
Gender (male/female)	8/8	6/2	0.388
Age (years)	19.2 ± 8.8		
(2–37)	34.5 ± 24.7		
(13–72)	0.264		
Location			0.234
Frontal lobe	1	1	
Temporal lobe	3	5	
Occipital lobe	2	1	
Parietal lobe	1	1	
Suprasellar region	5		
Dorsal thalamus			
Lateral ventricle	3		
	1		
Radiological pattern			1.000
Cystic-nodule	7	4	
Predominantly solid	9	4	
Peritumoral edema-no. (%)	9 (56.25%)	5 (62.50%)	1.000
Marked enhancement-no. (%)	10 (62.5%)	7 (87.50%)	1.000
“Dural tail” sign-no. (%)	1 (6.25%)	2 (22.22%)	0.249
Inner table scalloping-no. (%)	4 (25%)	3 (37.5)	0.647

*PA* pilocytic astrocytoma, *PXA* pleomorphic xanthoastrocytoma

Data in parentheses indicate the number of corresponding patients

**Fig. 1:**
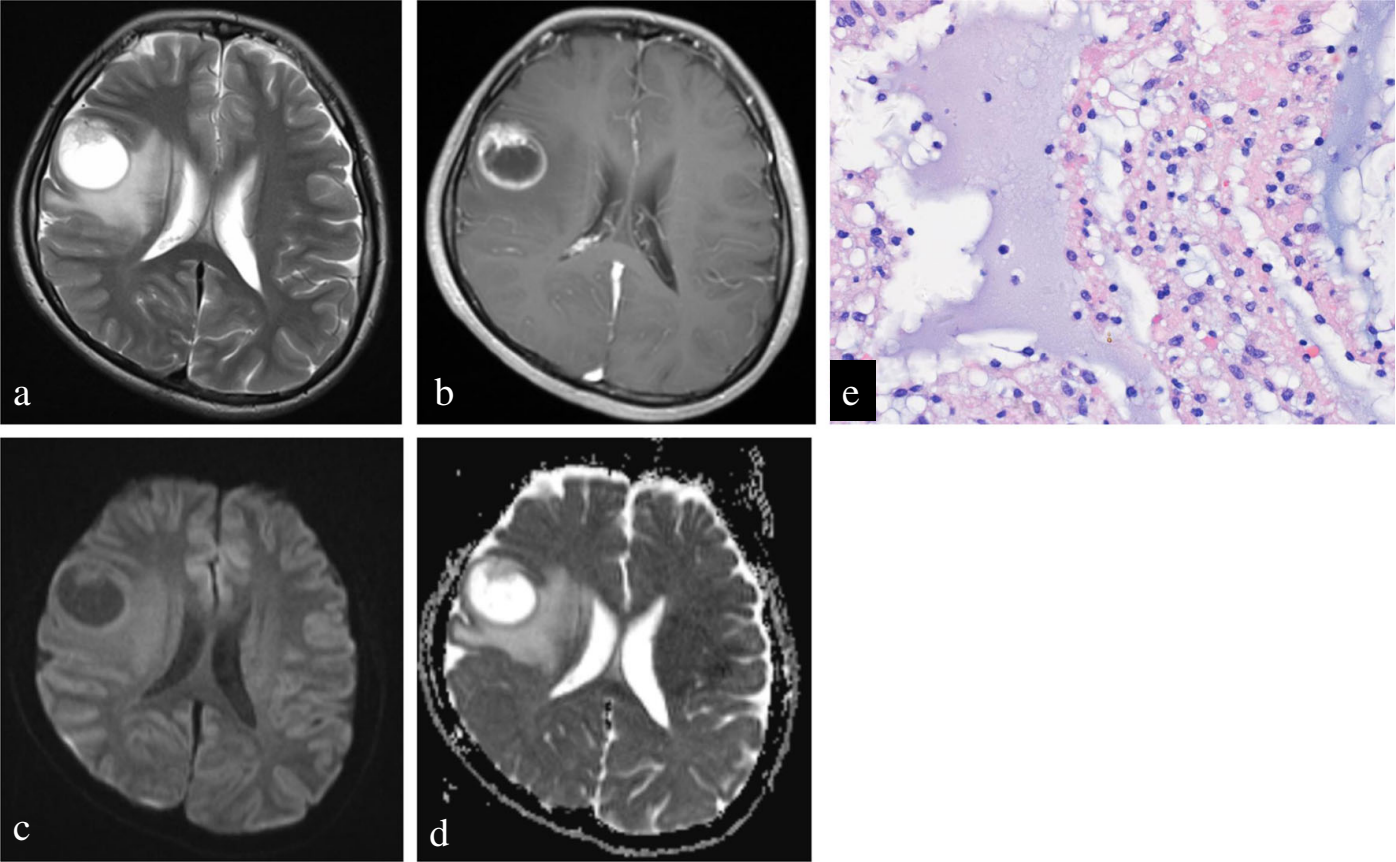
Right frontal pilocytic astrocytoma in a 16-year-old woman. **a** Preoperative axial T2-weighted image shows a cystic mass with a mural nodule. **b** Contrast-enhanced axial T1WI shows intense enhancement of the mural nodule and cystic wall. **c**, **d** A corresponding axial diffusion-weighted image and ADC map show that the contrast-enhancing solid nodule of tumor shows a moderately increased diffusion compared with the normal-appearing white matter (ADC_mean_ = 1621 × 10^−6^ mm^2^/s). **e** The tumor shows a biphasic appearance in a loosely arranged myxoid background (original magnification, × 400; hematoxylin-eosin stain)

**Fig. 2:**
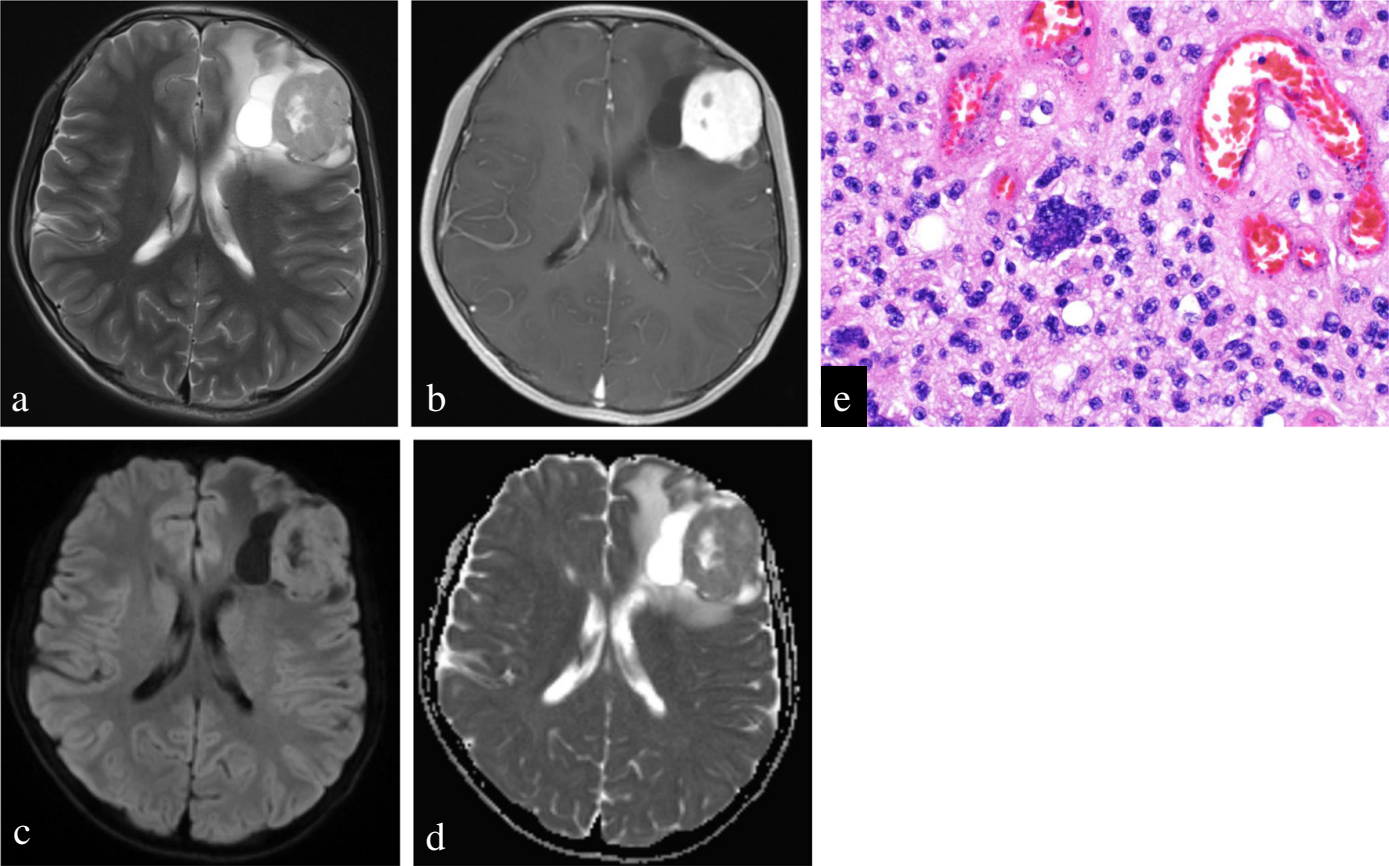
Left frontal pleomorphic xanthoastrocytoma in a 13-year-old man. **a** Preoperative axial T2-weighted image shows a cystic mass with a large solid component. **b** Contrast-enhanced axial T1WI shows intense enhancement of the solid component. **c**, **d** A corresponding axial diffusion-weighted image and ADC map show that the contrast-enhancing solid component of tumor shows a slightly increased diffusion compared with the normal-appearing white matter (ADC_mean_ = 1135 × 10^−6^ mm^2^/s). **e** The tumor shows densely cellular glial elements, including a large multinucleated xanthomatous cell with foamy cytoplasm (original magnification, × 400; hematoxylin-eosin stain)

The ADC parameters including ADC_mean_ values and ADC_min_ values calculated for supratentorial PA, lobar PA, and PXA are shown in Table 2. On DWI, the signal intensity in the contrast-enhancing regions of supratentorial PA was hyperintense (*n* = 4), isointense (*n* = 10), and hypointense (*n* = 2) relative to the contralateral normal-appearing white matter. Conversely, the signal intensity of PXA was hyperintense (*n* = 6), isointense (*n* = 2), and hypointense (*n* = 0). The signal intensity in the solid contrast-enhancing portions of PA was significantly higher than that of PXA (*P* = 0.02). Interobserver agreement of two readers for the semiquantitative analysis of all ADC parameters was excellent (ICC for ADC_mean_, 0.97; for ADC_min_, 0.78). Both ADC_mean_ values (1542 ± 186 vs 1084 ± 201 × 10^−6^ mm^2^/s; *P* < 0.001) and ADC_min_ values (1355 ± 183 vs 988 ± 180 × 10^−6^ mm^2^/s; *P* < 0.001) were significantly higher in supratentorial PA compared with PXA, respectively. In addition, Both ADC_mean_ values (1517 ± 218 vs 1084 ± 201 × 10^−6^ mm^2^/s; *P* < 0.001) and ADC_min_ values (1345 ± 322 vs 988 ± 180 × 10^−6^ mm^2^/s; *P* < 0.001) were also significantly higher in lobar PA compared with PXA, respectively. The results of the ROC curve analysis are given in Table 3. ROC curve analysis indicated that ADC_mean_ values seemed to account for the higher AUC (0.977) in differentiating supratentorial PA from PXA, with a cut-off value of 1189.8 × 10^−6^ mm^2^/s and sensitivity and specificity of 93.8 and 100%, respectively. But there were no significant differences in AUC between ADC_mean_ and ADC_min_ for differentiating supratentorial PA from PXA using *Z* test (*Z* = 1.702, *P* = 0.09). The ADC_mean_ values were also useful for discriminating lobar PA from PXA (AUC, 0.946) with a cut-off value of 1189.8 × 10^−6^ mm^2^/s (sensitivity, 85.7%; specificity, 100%). The decision plot for ROC analysis is shown in Supplementary Figs. 1–4. Representative cases of supratentorial PA and PXA mimicking each other are shown in Figs. 1 and 2.

**Table 2:** Differences of histogram parameters in supratentorial PA, lobar PA, and PXA (mean ± standard deviation)

Parameter (×10^−6^ mm^2^/s)	Supratentorial PA (*n* = 16)	Lobar PA (*n* = 7)	PXA (*n* = 8)	^a^ *P*	^b^ *P*
ADC_mean_	1542 ± 186	1517 ± 218	1084 ± 201	< 0.001	< 0.001
ADC_min_	1355 ± 183	1345 ± 322	988 ± 180	< 0.001	0.001

*PA* pilocytic astrocytoma, *PXA* pleomorphic xanthoastrocytoma

^a^*P* value of compared results of all supratentorial PA and PXA using unpaired Student’s *t* test. ^b^*P* value of compared results of a subgroup of lobar PA and PXA

**Table 3:** Diagnostic performance of ADC parameters for differentiating PA from PXA

parameter	TV (×10^−6^ mm^2^/s)	Sensitivity	Specificity	PPV	NPV	Accuracy	AUC
^a^ADC_mean_	1189.8	93.8%	100%	100%	88.9%	95.8%	0.977
^a^ADC_min_	1063.5	75.0%	87.5%	92.3%	63.6%	79.2%	0.820
^b^ADC_mean_	1189.8	85.7%	100%	100%	88.9%	93.3%	0.946
^b^ADC_min_	1063.5	85.7%	87.5%	85.7%	87.5%	86.7%	0.839

*TV* threshold value, *PPV* indicates positive predictive value, *NPV* negative predictive value, *AUC* area under the curve

^a^Comparison of all supratentorial PA and PXA. ^b^Comparison of lobar PA and PXA

## Discussion

In this study, we used ADC values derived from DWI to discriminate supratentorial PA and PXA, which is frequently indistinguishable with conventional MRI. Our preliminary results suggested that both ADC_mean_ and ADC_min_ values based on contrast-enhancing portions of the tumor in supratentorial PA group were significantly higher than those in PXA group.

Both PA and PXA belong to low-grade astrocytoma and gross total resection without adjuvant treatment including radiotherapy or chemotherapy is considered as the preferred treatment. However, the surgical planning of supratentorial cystic PA may slightly differ from cystic PXA. The surgical procedures for removal of cystic components of PA and PXA include radical resection of cyst wall, biopsy of cyst wall, or no resection at all. Previous studies have reported that the cyst wall of PA was free from tumor tissues even if it was enhanced on MRI [19], and removal of cyst did not improve patients’ survival in the series of 51 patients with cystic PA [20]. Furthermore, in clinical practice, radical removal of large cyst wall within supratentorial brain tumors may worsen motor deficit and increase the incidence of seizures [19]. Thus, a more conservative approach, leaving cyst walls intact, may be recommended for PA associated with large cysts. Nevertheless, Sakamoto et al. [21] showed that the cyst wall of PXA reflected neoplastic tissues, and tumors of PXA could recur from the cyst wall if tumor removal without resection of the cyst wall was implemented. In addition, PXA shows a strong intrinsic tendency to recur and undergo malignant transformation, especially if incomplete resection. Thus, radical tumor removal with resection of the cyst wall may be needed immediately for PXA to decrease postoperative recurrence incidence and long-term follow-up with repeated MRI is mandatory to detect tumor progression. A growing body of literature suggests that BRAF V600E mutation is a potentially targetable genetic abnormality in pediatric low-grade glioma, which is found frequently in PXA and less frequently in PA [22]. It has been demonstrated that PXA can be successfully treated with targeted BRAF mutations inhibitors in a small case series [23]. Therefore, the differentiation of PXA from PA may guide the targeted gene therapy in the future.

PA typically occurs in the cerebellum, whereas the overwhelming majority of PXA arises from the supratentorial brain. With the typical location and appearance of a well-delineated cerebellar cystic mass with mural nodule, the diagnosis of PA is easy. However, when located in the supratentorial region, PA shares its typical “a supratentorial cystic mass with enhancing mural nodule” appearance with PXA [6]. As presented in the previous studies, this typical MR imaging feature was found in 43.8% of the supratentorial PA and in 50.0% of PXA in our study [1, 24–27]. In addition, the involvement of the adjacent leptomeninges (dural tail sign) has been reported to be a characteristic feature of PXA [26], which may be helpful in differentiating PXA from supratentorial PA. Lim et al. [28] reported the involvement of the adjacent leptomeninges in 12 of 22 patients with PXA. Whereas in the study by Crespo-Rodríguez et al. [26], only 3 of the 14 patients demonstrated enhancement of the adjacent leptomeninges. In this study, we also found that only 2 of the 7 PXAs demonstrated the “dural tail sign,” consistent with the previous study by Crespo-Rodríguez et al. [26]. And our results showed that involvement of the adjacent leptomeninges was not a reliable MR feature for differentiating PXA from PA. Therefore, when a supratentorial cystic mass with solid-enhancing nodule is demonstrated on conventional MR images, there is less certainty as to whether the tumor is a supratentorial PA or a PXA.

DWI is a reliable and practicable MR technique and has been widely used to evaluate intracranial tumors [11–13, 15]. There are only a few studies assessing DWI in PXA, all of which are anecdotal case reports or case series in the literature [29, 30]. Moore et al. [29] demonstrated in a case series that the mean ADC values were 912 ± 219 × 10^−6^ mm^2^/s in the 7 PXA tumors. In the present study, we found that the signal intensity in the contrast-enhancing areas of PXA tended to be hyperintense or isointense relative to normal white matter on DWI. Furthermore, the mean ADC values of PXA in this study were comparable to previous findings [29, 30]. Several studies that have evaluated ADC values in PA have compared them with high-grade glioma, medulloblastoma, and ependymomas [11, 31–33]. Previous studies of PA have demonstrated moderately high ADC values in PA (1534–1688 × 10^−6^ mm^2^/s) [11, 30, 31]. Our study showed that supratentorial PA demonstrated a moderately increased diffusion with ADC_mean_ of 1542 ± 186 × 10^−6^ mm^2^/s, which was consistent with that in previous studies [11, 30, 34]. However, to the best of our knowledge, the usefulness of ADC values derived from DWI in differentiating supratentorial PA from PXA has not been investigated previously. In a study of brain tumors, Yamasaki et al. [30] reported that the ADC values were increased (1659 ± 260 × 10^−6^ mm^2^/s) in 3 patients with PA, while 1 PXA had relatively lower ADC value of 1009 × 10^−6^ mm^2^/s (without statistical analysis). In this study, we found that all ADC values of supratentorial PA were significantly higher than those of PXA. Furthermore, we also performed a subgroup analysis of lobar PA and found that all ADC values of lobar PA were also significantly higher than those of PXA. This finding could be explained by different histopathological features of PA and PXA. Histologically, PA is a tumor of low-moderate cellularity within markedly loose myxoid background [35], whereas PXA is a hypercellular tumor composed of pleomorphic cells with mesenchymal-like morphology [36]. As previously reported [37], ADC values were inversely related to tumor cellular attenuation and tumor cell nucleus-to-cytoplasm ratio in terms of water molecules diffusivity within brain tumors. The association of higher ADC values of PA may reflect the lower cell density compared with PXA. In addition to the high cellularity, the tumor cells of PXA are characteristically large and multinucleate with a relatively high nucleus-to-cytoplasm ratio, which may restrict movement of water molecules [36, 38]. Taken together, we may postulate that the lower ADC values found in PXA strongly suggest an increased tumor cell density and a higher nucleus-to-cytoplasm ratio in PXA tumors compared with supratentorial PA.

Recently, a histogram analysis based on whole-tumor has been used to indicate the heterogeneity of high-grade glioma and minimize ROI sampling errors [14, 39]. However, Xu et al. [39] suggested that the whole-tumor histogram method in assessing glioma did not yield higher interobserver agreement and better diagnostic performance than does the single-slice methods based on the minimum ADC value and took longer. In addition, the low-grade glioma including PA and PXA showed relatively less heterogeneity of tumor cellularity compared with high-grade glioma. Thus, we used single-slice measurements to indicate the diffusivity of water molecules in these two tumor types and shorter analysis time. Our results showed that both ADC_mean_ values and ADC_min_ values were significant for the contrast-enhancing portion of the tumor to discriminate supratentorial PA from PXA. Although ROC classification could only be used to illustrate the performance of a binary classifier system and AUC from ROC may be misleading if the ROC curves of two diagnostic tests intersect, ROC analysis is still a widely accepted method for evaluating the diagnostic accuracy of radiological tests [40]. In this study, we performed ROC analysis based on a binary comparison of PXA and PA, and ROC curve of ADC values does not intersect in our study. Our preliminary results showed that the accuracy levels were extremely high for ADC_mean_ values in distinguishing supratentorial PA from PXA (accuracy level, 95.8%), as well as in distinguishing lobar PA form PXA (accuracy level, 93.3%). Therefore, our findings suggest that DWI with ADC values may aid in the differential diagnosis of supratentorial PA and PXA, which is frequently challenging in clinical practice.

Besides the intrinsic limitations of the retrospective study, several other potential limitations of this study should be mentioned. Firstly, the number of the patients with PXA was rather small. Future investigations that include more patients are recommended to strengthen the statistical power. Secondly, because ADC values were not used to guide the biopsy in this retrospective study, it was not possible to evaluate the correlations between regions of lower ADC in PXA and hypercellularity point to point. These correlations should be performed in our future study. Thirdly, we could not excluded the presence of tiny hemorrhage within the tumor that may generate susceptibility blooming, hence influencing DWI evaluation, though there was no noticeable evidence of hemorrhage on conventional MRI. Fourthly, the diagnostic accuracy of DWI in our study refers only to the binary comparison of supratentorial PA and PXA, which may indicate these results cannot be used among PA, PXA, and other supratentorial young adult contrast-enhancing lesion, such as gangliocytoma, ganglioglioma, and high-grade glioma. However, the purpose of this study was to differentiate supratentorial PA from PA, which may be distinguishable on conventional MRI. And several conventional radiological features may be indicative of other supratentorial adult contrast-enhancing lesion. For instance, the presence of intratumoral calcification was a consistent feature for gangliogliomas and gangliocytomas. And the infiltrative growth pattern, heterogeneous enhancement, and the presence of hemorrhage and necrosis should indicate high-grade gliomas. Finally, supratentorial PA and PXA are not amenable for a surgical excision despite of preoperative accurate differential diagnosis. However, the surgical strategies for cystic components of these two tumors may be different in order to reduce postoperative disability and mortality without increasing the recurrence rate. Further prospective studies are needed to confirm the value of differential diagnosis of these two tumors in guiding the surgical plans.

In conclusion, our results suggest that DWI is helpful in characterization and differentiation of supratentorial PA from PXA.

## Electronic supplementary material

ESM 1 (PDF 1160 kb)

## Abbreviations

PA: Pilocytic astrocytoma
WHO: World Health Organization
PXA: Pleomorphic xanthoastrocytoma
DWI: Diffusion-weighted imaging
ADC: Apparent diffusion coefficient
TR: Repetition time
TE: Echo time
FOV: Field of view
ROI: Region of interest
ADC_mean_: Mean ADC values
ADC_min_: Minimum ADC values
ROC: Receiver operating characteristic
AUC: Area under curve
ICC: Intraclass correlation test
