# Supplementary material for: Diagnostic accuracy of diffusion weighted imaging for differentiation of supratentorial pilocytic astrocytoma and pleomorphic xanthoastrocytoma
Source: Neuroradiology. 2018 May 24;60(7):725–33. doi: 10.1007/s00234-018-2036-y (PMC5996010; doi:10.1007/s00234-018-2036-y)
Supplement: Supplementary file 1 — (PDF 1160 kb) [file 234_2018_2036_MOESM1_ESM.pdf]

Criterion	Sensitivity	95% CI	Specificity	95% CI	+LR	-LR	+PV	95% CI	-PV	95% CI
≥547	100.00	79.4 - 100.0	0.00	0.0 - 36.9	1.00		66.7	44.7 - 84.4		
>547	100.00	79.4 - 100.0	12.50	0.3 - 52.7	1.14	0.00	69.6	47.1 - 86.8	100.0	2.5 - 100.0
>967.37	93.75	69.8 - 99.8	12.50	0.3 - 52.7	1.07	0.50	68.2	45.1 - 86.1	50.0	1.3 - 98.7
>1018	93.75	69.8 - 99.8	37.50	8.5 - 75.5	1.50	0.17	75.0	50.9 - 91.3	75.0	19.4 - 99.4
>1028.6	87.50	61.7 - 98.4	37.50	8.5 - 75.5	1.40	0.33	73.7	48.8 - 90.9	60.0	14.7 - 94.7
>1032.33	87.50	61.7 - 98.4	50.00	15.7 - 84.3	1.75	0.25	77.8	52.4 - 93.6	66.7	22.3 - 95.7
>1059.25	75.00	47.6 - 92.7	50.00	15.7 - 84.3	1.50	0.50	75.0	47.6 - 92.7	50.0	15.7 - 84.3
>1063.5	75.00	47.6 - 92.7	87.50	47.3 - 99.7	6.00	0.29	92.3	64.0 - 99.8	63.6	30.8 - 89.1
>1116	56.25	29.9 - 80.2	87.50	47.3 - 99.7	4.50	0.50	90.0	55.5 - 99.7	50.0	23.0 - 77.0
>1119.13	56.25	29.9 - 80.2	100.00	63.1 - 100.0		0.44	100.0	66.4 - 100.0	53.3	26.6 - 78.7
>1794.38	0.00	0.0 - 20.6	100.00	63.1 - 100.0		1.00			33.3	15.6 - 55.3

Supplementary Figure 1. The decision plot of ROC analysis of ADC_{min} values in differentiating supratentorial PA and PXA.

Criterion	Sensitivity	95% CI	Specificity	95% CI	+LR	-LR	+PV	95% CI	-PV	95% CI
≥697.91	100.00	79.4 - 100.0	0.00	0.0 - 36.9	1.00		66.7	44.7 - 84.4		
>1096.85	100.00	79.4 - 100.0	62.50	24.5 - 91.5	2.67	0.00	84.2	60.4 - 96.6	100.0	47.8 - 100.0
>1141.31	93.75	69.8 - 99.8	62.50	24.5 - 91.5	2.50	0.10	83.3	58.6 - 96.4	83.3	35.9 - 99.6
>1189.8	93.75	69.8 - 99.8	100.00	63.1 - 100.0		0.063	100.0	78.2 - 100.0	88.9	51.8 - 99.7
>1828.62	0.00	0.0 - 20.6	100.00	63.1 - 100.0		1.00			33.3	15.6 - 55.3

Supplementary Figure 2. The decision plot of ROC analysis of ADC_{mean} values in differentiating supratentorial PA and PXA.

Criterion	Sensitivity	95% CI	Specificity	95% CI	+LR	-LR	+PV	95% CI	-PV	95% CI
≥547	100.00	59.0 - 100.0	0.00	0.0 - 36.9	1.00		46.7	21.3 - 73.4		
>547	100.00	59.0 - 100.0	12.50	0.3 - 52.7	1.14	0.00	50.0	23.0 - 77.0	100.0	2.5 - 100.0
>967.37	85.71	42.1 - 99.6	12.50	0.3 - 52.7	0.98	1.14	46.2	19.2 - 74.9	50.0	1.3 - 98.7
>1063.5	85.71	42.1 - 99.6	87.50	47.3 - 99.7	6.86	0.16	85.7	42.1 - 99.6	87.5	47.3 - 99.7
>1116	57.14	18.4 - 90.1	87.50	47.3 - 99.7	4.57	0.49	80.0	28.4 - 99.5	70.0	34.8 - 93.3
>1119.13	57.14	18.4 - 90.1	100.00	63.1 - 100.0		0.43	100.0	39.8 - 100.0	72.7	39.0 - 94.0
>1794.38	0.00	0.0 - 41.0	100.00	63.1 - 100.0		1.00			53.3	26.6 - 78.7

Supplementary Figure 3. The decision plot of ROC analysis of ADC_{min} values in differentiating lobar PA and PXA.

Criterion	Sensitivity	95% CI	Specificity	95% CI	+LR	-LR	+PV	95% CI	-PV	95% CI
≥697.91	100.00	59.0 - 100.0	0.00	0.0 - 36.9	1.00		46.7	21.3 - 73.4		
>1096.85	100.00	59.0 - 100.0	62.50	24.5 - 91.5	2.67	0.00	70.0	34.8 - 93.3	100.0	47.8 - 100.0
>1141.31	85.71	42.1 - 99.6	62.50	24.5 - 91.5	2.29	0.23	66.7	29.9 - 92.5	83.3	35.9 - 99.6
>1189.8	85.71	42.1 - 99.6	100.00	63.1 - 100.0		0.14	100.0	54.1 - 100.0	88.9	51.8 - 99.7
>1828.62	0.00	0.0 - 41.0	100.00	63.1 - 100.0		1.00			53.3	26.6 - 78.7

Supplementary Figure 4. The decision plot of ROC analysis of ADC_{mean} values in differentiating lobar PA and PXA.
